
JOURNAL INFORMATION
==============================
NLM Title Abbreviation: ScientificWorldJournal
Journal ID: ScientificWorldJournal
NLM Journal ID: 101131163
Journal ID: TSWJ

The Scientific World Journal

ISSN: 2356-6140
EISSN: 1537-744X
Publisher: Wiley

ARTICLE INFORMATION
==============================
PMCID: PMC6084110
PMID: 30147542
DOI: 10.1100/tsw.2001.252
Article ID: 905941
Article version: 1
Subjects: Short Report

Role of Telomerase in Maintaining Hematopoietic Stem Cell Telomere Length during Replicative Stress and Aging

Manning Erin L. *
Van Zant Gary
University of Kentucky, Lexington, KY, USA
*Erin L. Manning: elmann1@pop.uky.edu
Publication date: 2001
Electronic publication date: 2001 Dec 19
Volume: 1
First page: 69
Last page: 69
Copyright: Copyright © 2001 Erin L. Manning and Gary Van Zant.
Copyright year: 2001
License: This is an open access article distributed under the Creative Commons Attribution License, which permits unrestricted use, distribution, and reproduction in any medium, provided the original work is properly cited.
License URL: https://creativecommons.org/licenses/by/3.0/

Funding: National Institutes of Health R01AG16653 National Institutes of Health 1T32AG242

==============================
Miami *Nature Biotechnology* Short Reports
*TheScientificWorld* (2001) 1, 67SR
ISSN 1532–2246; DOI 10.1100/TSW.2001.252

### ROLE OF TELOMERASE IN MAINTAINING HEMATOPOIETIC STEM CELL TELOMERE LENGTH DURING REPLICATIVE STRESS AND AGING

Erin L. Manning* and Gary Van Zant

Departments of Physiology and Internal Medicine, University of Kentucky
Markey Cancer Center CC415, 800 Rose Street, Lexington, KY 40536-0093

*elmann1@pop.uky.edu

**INTRODUCTION.** Telomeres and telomerase expression have been implicated in neoplastic progression of human tumors and in replicative senescence of cultured cells. However, their role in replicative senescence and aging of cell populations in vivo is less certain, despite the fact that human telomere length is age-related in rapidly dividing tissues such as hematopoietic cells (1). Given that relatively short-lived inbred mice have telomeres several times longer than humans, the relevance of telomere shortening to aging in mice is even more doubtful. Using a Flow cytometry-based fluorescence *in situ* hybridization (Flow-FISH) method of measuring telomere length in individual cells, we have studied the effects of aging and sublethal irradiation on hematopoietic cells of long-lived (C57BL/6) and short-lived (DBA/2) mouse strains.

**METHODS.** *Animals.* Female DBA/2 and C57BL/6 mice (ages 2 and ~20 months) were used.

*Flow FISH Telomere Length Analysis.* Fluorescence *in situ* hybridization with flow cytometry (Flow FISH) was done using the method of Lansdorp *et al*. (2)

*Telomerase Assay.* Telomerase activity was determined using the Boerhinger Mannheim Telomerase PCR ELISA kit based on the Telomeric Repeat Amplification Protocol (TRAP).

*Stem Cell Purification.* Stem cells (Sca-1+, c-kit+, and lin-) were purified from pooled bone marrow of 13-15 animals as previously described (3).

**RESULTS.** Telomere length of hematopoietic stem cells purified from bone marrow of young (2 mo.) animals of both strains showed a consistent strain difference where DBA/2 stem cells had ~30% longer telomeres than those of C57BL/6 mice. Stem cells purified from old (23 mo.) animals of both strains showed no detectable telomere shortening relative to young animals, and the strain difference was maintained. Thus aging had no demonstrable effect on stem cell telomere lengths in these strains. Aging, however, had a significant effect on telomerase activity in hematopoietic stem cells. In both strains, telomerase levels were elevated in stem cells purified from older animals. Because we found that telomere measurements of blood leukocytes accurately reflected telomere lengths of purified stem cells in young and old animals of both strains, we tested the effects of sublethal irradiation on hematopoietic recovery during which blood cells from mice could be repetitively sampled.

One month after irradiation (5 Gy), we found no detectable change in telomere lengths of young animals of either strain. However, in old animals of both strains, telomeres shortened dramatically. In C57BL/6 animals, telomeres shortened ~23% one month after 5 Gy ($P<0.05$). Old DBA/2 mice showed an even larger reduction of ~38% ($P<0.01$) in length despite the fact they were given 4 rather than 5Gy (5 Gy was fatal).

**DISCUSSION.** The dramatic reduction in telomere length following irradiation in old mice of both strains suggests either a diminished repair mechanism in old mice and/or accelerated telomere loss per cell division. We conclude that aging affects the ability of stem cells to maintain telomeres in vivo in the face of replicative stress. Higher levels of telomerase activity seen in older animals may act as a compensatory mechanism to maintain telomere length during normal aging, but may be insufficient to prevent telomere shortening in old animals subjected to replicative stress.

**ACKNOWLEDGEMENT.** This work was supported by National Institutes of Health Grants R01AG16653 (GVZ) and 1T32AG242 (ELM).

**REFERENCES.**

1. Rufer, N., Brummendorf, T.H., Kolvraa, S., Bischoff, C., Christensen, K., Wadsworth, L., Schulzer, M., and Lansdorp, P.M. (1999) J. Exp. Med. 190, 157-167
2. Rufer, N., Dragowska, W., Thornbury, G., Roosnek, E., and Lansdorp, P.M. (1998) Nat. Biotechnol. 16, 743-747
3. De Haan, G., Szilvassy, S.J., Meyerrose, T.E., Dontje, B., Grimes, B., and Van Zant, G. (2000) Blood 96, 1374-1379
